# Characterization and Strength Quality of the *Oryctolagus cuniculus* Leather Compared to *Oreochromis niloticus* Leather

**DOI:** 10.1155/2022/4561404

**Published:** 2022-10-14

**Authors:** Gislaine Gonçalves Oliveira, Eliane Gasparino, Leandro Dalcin Castilha, Nilton Garcia Marengoni, Elenice Souza dos Reis Goes, Fernanda Losi Alves de Almeida, Marcos Antonio Matiucci, Andresa Carla Feihrmann, Gabriela Hernandes Granzoto, Jaisa Casetta, Sandro de Vargas Schons, Jerônimo Vieira Dantas Filho, Maria Luiza Rodrigues de Souza

**Affiliations:** ^1^Universidade Estadual de Maringá (UEM), Programa de Pós-Graduação em Zootecnia, Maringá, PR, Brazil; ^2^Universidade Estadual do Oeste do Paraná (UNIOESTE), Programa de Pós-Graduação em Recursos Pesqueiros e Engenharia de Pesca, Marechal Cândido Rondon, PR, Brazil; ^3^Universidade Federal da Grande Dourados (UFGD), Engenharia de Aquicultura, Dourados, MS, Brazil; ^4^Universidade Federal de Rondônia (UNIR), Programa de Pós-Graduação em Ciências Ambientais, Rolim de Moura, RO, Brazil

## Abstract

This study aimed to compare the resistance of the *Oryctolagus cuniculus* L. (rabbit) and *Oreochromis niloticus* L. (Nile tilapia) skins, as well as to observe the design of the flower of these skins and the morphology of the dermis. Tilapia and rabbit skins were placed inside the same equipment (tannery machine) for the chromium salt tanning process. The flower design of the fish leather distinguishes it from the rabbit leather, the latter being constituted by the opening of the hair follicles and pores, while the fish leather is constituted by the presence of protective lamellae and insertion of the scales. The dermis of rabbit skin consists of thick bundles of collagen fibers arranged in all directions, which differs from the morphology observed in the dermis of fish skin. However, in the Nile tilapia skin dermis, overlapping and parallel layers of longitudinal collagen fiber bundles are observed, these layers are interspersed with fiber bundles crossing the sking surface (transversely), tying the fibers together and providing greater strength, which can be proven by the strength test. The fish leathers, despite having less thickness (1.0 mm), demonstrated significantly greater tensile strength (13.52 ± 1.86 N mm^−2^) and tear strength (53.85 ± 6.66 N mm^−2^) than rabbit leathers, that is, (8.98 ± 2.67 N mm^−2^) and (24.25 ± 4.34 N mm^−2^). However, rabbit leather demonstrated higher elasticity (109.97 ± 13.52%) compared to Nile tilapia leather (78.97 ± 8.40%). It can be concluded that although the rabbit leather is thicker due to the histological architecture of the dermis (thick bundles of collagen fibers arranged in all directions with no pattern of organization of collagen fibers), it shows less resistance than Nile tilapia leather, which demonstrates an organization of overlapping and parallel layers and intercalating collagen fiber bundles transversally to the surface, functioning as tendons for the swimming process. It is recommended to use a piece of fabric (lining) together with the fleshy side of the rabbit leather, to increase resistance when used in clothing and footwear, as these products require greater tensile strength. Thus, it minimizes this restriction for the use of rabbit leather in the aforementioned purposes.

## 1. Introduction

Among the animals for meat production, regardless of the species, the amount of by-product generated in the processing is extremely high [1]. Of these by-products, the skin shows a significant percentage, since it involves the entire body area of the animal, and for fish skin, such as *Oreochromis niloticus* L. (Nile tilapia), the corresponding value is 4.0 to 14% to body weight, depending on how the fish is filleted and the skin is removed [2]. This variation in percentages is due to the animal species, the filleting method, the filleter's dexterity, and the way the skin is removed (manually with or without the use of mechanical pliers). For *Oryctolagus cuniculus* L. (rabbit), the percentage of skin is 15 to 16% of the body weight of animals slaughtered at 70 days [3]. The form of skinning (removal of the animal's skin) also influences the useable area of the skin, as well as the cut made in the ventral region after slaughter, to open the skin for processing [4, 5].

Rabbit skin is a by-product that can be processed with or without hair to obtain, after a tanning process, a raw material with high softness, elasticity, and beauty, for its softness to the touch and with an extremely delicate flower design (surface of the skin without hair) [6, 7]. After the tanning process, the leather is transformed into a beautiful product when well-handled during rearing and slaughter and can be directed to the different tanning techniques, and the two results, with or without hair, are excellent in terms of final raw material, differentiating in the aspect of its applicability in clothing, mainly in the article [8]. However, these leathers are currently being discarded or underutilized due to the lack of proper tanning techniques, and preservation and storage systems [1, 4].

Based on the overview presented, it is understood the rabbit and Nile tilapia are animals that will be industrialized foods, the study of leather resistance is a way of optimizing the use of industrialization residues; therefore, it is a by-products quality science. To better know the quality of the leathers, it is very important to evaluate their resistance and compare them between species to know their characteristics, after the transformation of the skin into leather, with the possibility of use, especially in the confection of clothing, footwear, and products that require greater resistance for their application.

In face of assumptions, the aim of this study was to compare the resistance of *O. cuniculus* (rabbit) and *O. niloticus* (Nile tilapia) leather and to learn about the histological architecture of the dermal layer of these two species.

## 2. Material and Methods

**Figure 1:**
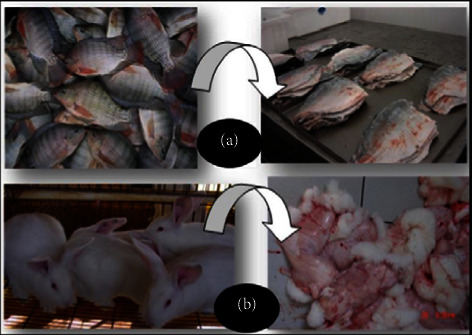
Rabbit and Nile tilapia skins are commonly discarded or underutilized. (a) Nile tilapia skins after skinning and (b) rabbit skins after skinning, with the hypodermic layer to the outside.

There were 40 skins of rabbits (*Oryctolagus cuniculus* Linnaeus, 1758) at 70 days of age (2,100 g) and 10 kg of skins of Nile tilapia (*Oreochromis niloticus* Linnaeus, 1758) with an average body weight of 700 g were used. The rabbit skins were obtained from disposal after the slaughter of animals from the Fazenda Experimental de Iguatemi (FEI) rabbit farm, which belongs to the Universidade Estadual de Maringá (UEM). The tilapia skins were obtained from the Smart Fish Company, Rolândia, PR, Brazil. The rabbit and Nile tilapia skins were taken to small and medium-sized skin processing laboratories at the FEI/UEM, Maringá, PR, Brazil. The skins were frozen (−18°C) until the moment of processing (Figure 1).

To better compare the quality of resistance between rabbit and Nile tilapia skins, we opted for the tanning process of the skins without hair, because for the permanence of the hair on the skin (furry) the process would be different from the one applied for the fish tanning. The steps responsible for removing this epidermal system are liming, deliming, and purging, which remove hairs or scales and opens up the fibrous tissue of the dermis, releases the interfibrillar material, and causes better intumescence of dermal structure, for the entry of chemicals [9].

To start the tanning process, the skins were placed in water at room temperature for thawing. After thawing, the rabbit skins were prepared for the tanning process by cutting out the unwanted parts (tail and genitalia) and opening the ventral region of the skin, therefore careful consideration was taken not to remove too much below the tail insertion, thus avoiding affecting the cut line and reducing the tanning skin area [5].

After defrosting, only the rabbit skins were submitted to fleshing (Figure 1), to with the purpose of removing the remains of meat and fat adhered to the skin [5]. For the tilapia skins, it was not necessary to perform the fleshing, since they were skins coming from a slaughterhouse that uses a machine to remove the skin from the filet. The skins come out without meat residues when the machine is well-calibrated.

Then, the rabbit skins were weighed in order to determine the percentage of chemicals to be used for the soaking stage. For the fish skins, in which the Nile tilapia was used, there was no need for clipping before processing; they were just weighed to include the weight along with those of rabbits for weighing the chemicals. The skins were placed inside the same equipment (tannery machine) and subjected to the same processing technique using the postdairy weight of the rabbit skins along with the weight of the fish skins as the basis.

The processing followed the methodology described by Castilla and Souza [5]. The steps that the fish and rabbit skins have undergone were soaking and liming (4% lime and 8% Dermaphel plus®, 0.5% surfactant), a step that was performed twice to remove all the rabbit hair (there was no need for tilapia skins because the scales come off very easily; however, the skins of both species remained in the same process to follow step by step processing defined for the rabbit skins). The process continued through the stages of deliming, purging, degreasing, pickling, tanning (6% chrome salts), neutralizing, retanning (2% Weibull®, 2% Syntac F®, 1% Tamol®), dyeing, fatliquoring (8% oil), drying, and fluffing.

For the skins to transform into leather, a period of four days was necessary, which was distributed among the different stages being mentioned above. After that, they were put on clotheslines for drying and softening, and the time required for this procedure depends on the ambient temperature. Fish leather dried faster due to its lower thickness compared to rabbit leather.

**Figure 2:**
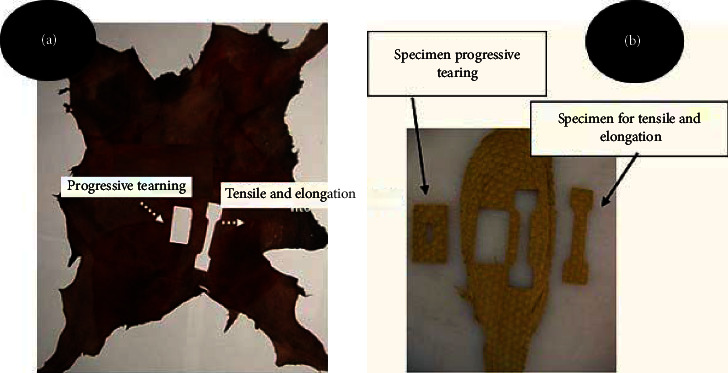
Position of specimen removal on rabbit (a) and Nile tilapia (b) leathers. Specimen for tensile and elongation; 1 = and specimen for progressive tearing test 2. Source: (a) Nascimento et al. [15] and (b) author.

After tanning the fish and rabbit skins, the specimens were removed with the help of a rocker [10] and then were taken to the laboratory under an acclimatized environment of around 23 ± 2°C and a relative humidity of 50 ± 5%, for 24 hours [11]. Thickness measurements [12] were determined for tensile strength and elongation calculations [13], and progressive tearing [14] was performed on the EMIC dynamometer, with a load spacing speed of 200 ± 20 mm min^−1^ and with the load cell at 200 kgf. The sections of specimens were cut longitudinally along the length of the body (cephalotail axis) for both species, in order to perform the strength tests (Figure 2).

Rabbit and Nile tilapia skin samples from the dorsal region were collected and fixed in Bouin for 24 hours. After this period, the samples were submitted to routine histological processing for paraffin embedding. Histological sections with a thickness of 5 µm were obtained on a microtome and were stained with Masson's trichrome. The histological slides were analyzed under a light microscope and were photomicrographed under the AXIOSKOP-ZEISS photomicroscope, Quanta™ 250 FEI, USA.

Samples of the dorsal region of the rabbit skin were collected for analysis under the scanning electron microscope (SEM). The fragments were fixed in 2.5% buffered glutaraldehyde and postfixed in 1% osmium tetroxide for two hours. They were then washed in phosphate buffer, dehydrated in a series of ethanol of increasing concentrations, and dried to a critical point with CO_2_. Specimens were metallized with gold-palladium ions and electrographed with JEOL-JSM 5410.

An entirely randomized design with two treatments (rabbit and Nile tilapia leather) was used, with 20 repetitions per treatment. The experimental unit was leather. The results of resistance tests were submitted to variance analysis, and the means were compared using the F test, at a 5% probability level (*α* = 0.05). Data were analyzed using the Statistical Analysis System (SAS) version 2010, a computer program.

## 3. Results

**Figure 3:**
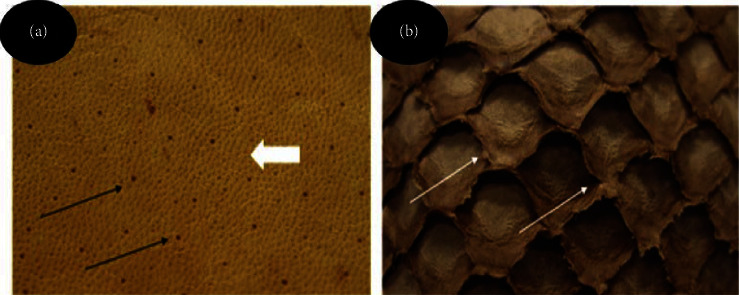
Flower design of rabbit (a) and Nile tilapia (b) leather. (a) The surface of the leather, after hair removal, performed in the tanning process, shows the pores (white arrow) and hair follicle openings (black arrow), from which the hairs project; (b) protective lamellae and insertion of the scales. The white arrows indicate the point of union between these lamellae and the dotted indicates the opening of the lamellae.

In Figure 3(a) (rabbit skin), it is possible to observe the presence of pores and the openings of hair follicles after hair removal, due to the action of the liming stage in the tanning process. In Figure 3(b) (Nile tilapia skin), one can see the opening and length of the protective lamellae, as well as their insertion, where the scales were inserted before the removal of the fish skin in the tanning process. The opening and length of the lamellae increase with the growth of the fish.

**Figure 4:**
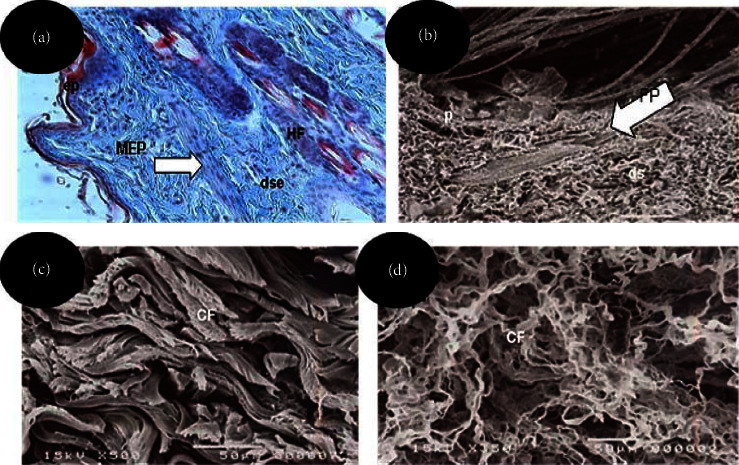
(a) Photomicrograph of a histological section of rabbit leather, illustrating the epidermis (ep) and superficial dermis (dse), containing the hair erector muscle (HEM) and hair follicle (HF). Staining: Masson's trichrome. Objective lens: 20X; (b) scanning electron micrographs illustrating the superficial dermis (ds), the hair follicle (FP), and hair (p) of various thicknesses found in the skin before the hair removal tanning process; (c, d) Scanning electrographs illustrating the distribution of collagen fibers (CF) in the dermis of rabbit leather; (d) loose collagen fibers on the flesh side after fluffing.

There are different types of hairs on rabbit skin and they are classified by size, thickness, and quantity. In Figure 4(b), we can see thicker hairs, which are the guide or guard hairs, and they are distributed over the skin in less quantity and are longer. When they are removed in the tanning process, specifically in the liming stage, the holes from where these hairs emerged can be seen in the leather, because they are larger (Figure 3(a), black arrow). There are the intermediate hairs which determine the typical coat color of each breed of rabbit and are smaller in number and finer compared to guide hairs. There are also shorter hairs which are thinner and more numerous than the intermediate hairs which are known as the dregs. When the intermediate hairs and dregs are removed, one can observe, after tanning, the fine pores (Figure 3(a)-white arrow ) evenly distributed over the entire surface of the leather.

In Figure 3(a), a photomicrograph of a histological section of rabbit skin without hair removal is observed, and it shows the epidermal layer consisting of a keratinized stratified sidewalk epithelium composed of 3 to 4 layers of cells. Below the epidermis lies the superficial dermal layer, composed of loose connective tissue containing fibroblasts and thin collagen fibers stained in blue by Masson's trichrome. In the deep dermis, a predominance of thick bundles of collagen fibers is observed which are arranged in all directions, characterizing a nonmodeled dense connective tissue.

Rabbit and fish skins are composed of two layers: the outer layer or epidermis (Figures 4(a) and 4(b)) and the underlying layer, called the dermis (Figure 4(c)). Below the dermis lies the hypodermis or subcutaneous layer (Figure 4(d)), which consists of fatty tissue and is close to the muscles. In tanning, for leather production, the epidermal layer is removed, while for fur tanning it should remain, and it is important that the hairs must not be removed, broken, or tangled. For the scaly fish skin tanning process, this layer will always be removed for the removal of the scales by the tanning process.

Scanning electron microscopy of the rabbit skin without hair removal (Figure 4(b)) shows the superficial dermis, the presence of hair follicles and hairs of different thicknesses before the tanning process. After tanning, thick and intertwined collagen fibers are observed in the dermis which are arranged in various orientations after softening of the leathers (Figure 4(c)) and it loosens on the flesh side of the skin after fluffing (Figure 4(d)).

**Figure 5:**
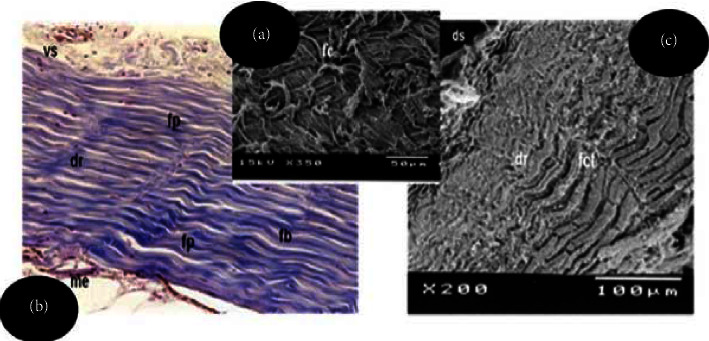
(a) Photomicrograph of histological section Nile tilapia leather illustrating the superficial dermis containing blood vessels (vs) and deep dermis formed by overlapping layers of parallel and longitudinal collagen fibers (fp). In the hypodermal layer, the presence of melanophores (me) is observed. Staining: Masson's trichrome. Objective lens: 20x; (b) scanning electron micrograph of the superficial dermis (ds) and deep dermis (dp), in which the overlapping layers of parallel collagen fibers interspersed by the transverse fibers (fct) are observed. Magnitude: 200x. Bar: 100 *μ*m; (c) scanning electron micrograph showing the interweaving of the collagen fibers (fc) of the superficial dermis. Magnitude: 350x. Bar: 50 *μ*m.

Below the compact dermis, a narrow layer of loose connective tissue with fine collagen fibers and fibroblasts is observed, containing adipocytes and a large number of melanocytes (Figure 5(a)), characterizing the hypodermic layer (Figures 5(b) and 5(c)).

The leathers analyzed in this study demonstrated significant differences (*P* < 0.05) for thickness (Table 1), with rabbit leather being thicker (1.43 mm) compared to Nile tilapia leather (1.00 mm).

The leathers demonstrated significant differences (*P* < 0.05) for tensile strength and progressive tearing tests (Tables 1 and 2). Nile tilapia leathers showed significantly higher resistance (*P* < 0.05) to tearing (53.85 ± 6.66 N mm^−1^) and traction (13.52 ± 1.86 N mm^−2^) when compared to rabbit leathers resistance to tearing (24.25 ± 4.34 N mm^−1^) and traction (8.98 ± 2.67 N mm^−2^). However, for elongation, rabbit leather demonstrated higher elasticity (109.97 ± 13.52%) than that of Nile tilapia leather (78.97 ± 8.40%) (Table 2).

## 4. Discussion

### 4.1. Morphology of Rabbit and Nile Tilapia Skins

The collagen fibers are composed of collagen fibrils, which are visible only through electron microscopy. Blood vessels, nerves, sebaceous glands, and erector muscle are found in this dermal layer [16]. With the tanning process, these structures are removed and this dermal layer is considered the most important for tanning, as these collagen fiber (CF) bundles react with the tanners in the process of transforming the skin into leather [17].

Unlike the rabbit dermis morphology, which presents collagen fibers arranged in various directions, in the fish skin, the two layers of collagen fibers are distributed in the thickness of the dermis. Corroborating with the description of Souza et al. [9], the superficial dermis is composed of loose connective tissue consisting of fine collagen fibers, fibroblasts, lymphocytes, and blood vessels. The deep or compact dermis of the fish skin, on other hand, is constituted by a modeled dense connective tissue, in which separate and overlapping layers of parallel collagen fiber bundles predominate in longitudinal orientation, interspersed by bundles of perpendicular collagen fibers in smaller quantity and visualized in transversal cut, that is, crossing the skin surface transversally in order to bind the fibers to provide greater resistance [9]. A narrow layer of loose connective tissue with fine collagen fibers and fibroblasts is observed below the compact dermis, where the presence of adipocytes and a large number of melanocytes, characterizes the hypodermic layer. Oznurlei et al. [18] and Zapletal et al. [19] also state that this layer is rich in adipocytes.

### 4.2. Comparison of the Strength Quality of Rabbit and Nile Tilapia Leathers

The rabbit leathers were thicker (1.43 mm) compared to Nile tilapia leather (1.00 mm). In contrast, Souza et al. [20] evaluated the quality of rabbit leathers compared to those of Nile tilapia and reported that the leathers did not demonstrate a significant difference in thickness (Nile tilapia = 0.44 mm and rabbit = 0.49 mm). Nevertheless, the authors used leathers from rabbits of 70 days of age at slaughter, with 1980 g and Nile tilapia with 500 g of body weight. Perhaps this thickness is related to the position (anterior or posterior) of the removal of the specimen, associated with the tanning technique applied and the size or weight of the animals. The authors used 3% sodium sulfide, which is a more aggressive product compared to the 6% Dermaples plus® used in the liming of this experiment; thus, there was a reduction in skin thickness reported by Souza et al. [20] and Matteucci et al. [21]. Another point to evaluate is that in this experiment the fish and rabbits demonstrated greater body weight when slaughtered, providing greater thickness to the leather.

When addressing the tanning technique, it may refer to any of the stages of the process or chemicals used, as well as their concentration. Among the products, the use of chromium salts provides a lower thickness compared to those tanned with vegetable tannin [8, 22], and the use of liming agents, as they are responsible for opening and eliminating the interfibrillar material, also reduces the thickness of the leather [23]. The purging step interferes directly with the thickness of the leather, because in this step the proteolytic enzymes act on undesirable proteins, to promote better opening and help penetration of other chemicals, thus reducing thickness [9]. To evidence this information, Hoch et al. [22] analyzed leathers from rabbits slaughtered at 70 days and reported that they demonstrated a thickness ranging from 0.49 to 0.87 mm, depending on tanning agents used in different tanning techniques. The authors mention that the leathers that were tanned with vegetable tannin showed significantly the highest thickness (0.87 mm), while those tanned with synthetic tannin resulted in the lowest thickness (0.49 mm). On the other hand, Fuck et al. [24], conducted another type of research and observed no significant difference in the thickness of leathers when they used tanning agents with different concentrations, whose thickness values ranged from 0.92 to 1.10 mm.

However, this is possible because, according to Vieira et al. [25] and Pradeep et al. [26], depending on the proportions and combinations of vegetable and synthetic tannins used, they do not interfere with the leather thickness. However, these authors also observed that Nile tilapia leather submitted to tanning with 5% vegetable tannin combined with 5% synthetic tannin demonstrated greater thickness (0.73 mm) when compared to leather tanned with 12% synthetic tannin (0.59 mm). Therefore, even with a lower concentration of tanning agents, when combined with vegetable tannin, it provided greater thickness to the leather [26].

Another factor that, according to Franco [27] and Patel et al. [28], can interfere with leather thickness is the tanning process time. The authors reported that rabbit leathers submitted to tanning technique with continuous time (shorter tanning time) demonstrated significantly greater thickness. The authors stated that the chemicals used did not fully react with the collagen fibers, leaving overlapping layers of the reagents used (1.063 mm). On the other hand, when rest intervals were used during certain stages of the tanning process, and specifically after basification, it provided a longer reaction time of the chemicals with the collagen fibers, and thus, the leathers showed less thickness (0.89 mm). According to Hoinacki [16], it is in this resting time, that the reticulation process occurs, where the chemical products (mainly the tanning agents) keep reacting with the collagen fibers.

As for size, body weight, or age, Santos et al. [29] evaluated Nile tilapia skins with body weights ranging from 600 to 1000 g, with a thickness of 0.77 to 0.98 mm. Authors reported that increasing weight of the fish at slaughter will increase the skin thickness, and this is explained by the positive linear equation *Y* = 0.745990 + 0.044832*X* (*R*^2^ = 88.27%). The same can be observed in rabbit leathers. Animals slaughtered at an older age (breeders) showed greater thickness than rabbit leathers slaughtered at 70 days of age [15]. Animals slaughtered at 400 days showed higher tensile strength (10.34 N mm^−2^), and in the transversal direction of leather, the highest elasticity (71.09%). At 70 days, the leather demonstrated higher elasticity in the transversal direction, regardless of whether the leather came from a male or a female. Therefore, the thickness of the leather is related to the size, weight, or age of the animals, the animal species, the position of removal of the leather specimen, and the tanning technique applied, as well as the strength quality of the leather [30].

When considering fish leather submitted to the same processing conditions in this study, the justification for the statistically significant difference in the tensile strength and progressive tearing tests is due to the fish's own skin differential structure because of flower design. Nile tilapia leathers have a superficial dermal layer that is loose and another one that is compact, in which there are overlapping layers of collagen fibers that are parallel, in opposite directions, and interwoven from space to space in the transverse direction to the leather surface. In this way, the collagen fibers are well interwoven, providing a high binding of the fibers [9]. Meanwhile, in rabbit skins, there are no regular patterns of arrangement and orientation of the collagen fibers. But regardless of the animal species, the skins have collagen fiber bundles, composed of fibrils.

The arrangement of collagen fibers in the deep dermis of fish resembles that of mammalian tendons (dense modeled connective tissue whose fibers resist tensile forces always in the same direction which explains why these fibers are arranged in parallel). Thus, their properties are similar to those of tendons to improve locomotion, as they perform a function analogous to tendons by transmitting the force of contraction to leather and thus allowing undulation when swimming (locomotion) [31]. As for the deep dermis in rabbit leather, the organization of the fibers in various directions and orientations allows them to resist the tension forces that are also exerted on leather in various directions, but its function is very different from what is physiologically required for Nile tilapia leather, and in other researches, the morphology of the moderate dense connective tissue is always directly related to its function [32]. Thus, one can infer that the deep dermis of mammalian skin in general is less resistant than that of a tendon, precisely because of this type of organization of collagen fibers [9].

The tensile response of Nile tilapia leather stretched in the anteroposterior direction is generated by two main features, the histological and the mechanisms of the compact dermis, where the angle of the fibers make up the collagen cross-ply and crimp of the collagen fibers [33]. These structural features of the dermis produce the tendon-like response of leathers, including an initial complacent phase and a subsequent stiffening phase that can result in the storage and recovery of elastic deformation energy. Then, these collagen fibers promote muscle contraction, providing the skin with an external tendinous function, occurring the muscular contraction of the fish to obtain the ripple movement to perform locomotion (swimming) [33]. However, to complement this, the structural and tensile properties of the dermis vary along the anteroposterior axis of the fish itself, because in the dorsal and tail regions, the leather promotes stiffer and stronger responses, suggesting differences in function for the different regions of the fish [34]. It can be inferred that there are structural differences in the arrangement and orientation of collagen fibers depending on the different regions of the fish skin to efficiently exert its locomotion process [35]. As leathers exhibit light weight, flexibility, high protective capacity (or puncture resistance), and energy storage and recovery capacity (or tendon-like properties), Szewciw and Barthelat [33] reported that the compact stratum of the fish dermis consists of around 30 layers of highly ordered collagen fibers of alternating caudodorsal and caudoventral direction, with fiber angles of 60.51 ± 7.07° (*n* = 30) and 57.58 ± 6.92° (*n* = 30), respectively, which provides fish movement, and it shows a decrease in fiber angle from the head region toward the tail region of the fish. In this process, studies stratum of the dermis fish compact showed an energy storage mechanism that acts similarly to a tendon, which also help in the locomotion of fish. However, the mechanism needs to store and release enough strain energy to facilitate muscle contraction. Therefore, the leather flexes to the ripples close to the fins. This information justifies the question in terms of the greater resistance of leathers compared to rabbit leathers.

Souza et al. [36] also observed that Nile tilapia leather showed higher tensile strength (20.93 N mm^−2^) and progressive tearing (34.04 N mm^−1^) compared to rabbit leather (13.91 N mm^−2^ and 17.36 N mm^−1^, respectively). However, the results demonstrated by the authors are lower than those obtained in this study (Tables 1 and 2) deducing the same parameters discussed for the thickness of the leathers regarding the tanning process, distribution, and organization of the collagen fibers.

The maximum shape applied in the progressive tearing test for fish leather was significantly higher than that of rabbit leather (Table 1), demonstrating in this test that to continue the tearing, there was the need for an amount of force in Newton which was significantly higher than that of rabbit leather. However, the maximum breaking force applied in the traction test did not show a significant difference between leathers (Table 2). This is probably due to the distribution of the fine collagen fibers present in the dermis. In this study, we did not quantify the thick collagen fibers (type I) and the thin collagen fibers (type III). However, Corrêa et al. [37] quantified the collagen fibers of three fish species (Nile tilapia, *Micropogonias undulates* and *Merluccius merluccius*). The authors reported that *M. undulates* and *M. merluccius* have the same proportions of fine and thick collagen fibers, while Nile tilapia has the highest proportion of thick fibers. Therefore, the authors concluded that the yellow hake leather demonstrated higher resistance for all parameters evaluated (tensile = 24.81 N mm^−2^; elongation = 83.24%, and tear = 95.87 N mm^−1^), due to the histological arrangement of the collagen fibers, where they are intercalated in such a way that the thin fibers tie the thick ones very well. Nevertheless, even with lower resistance, the Nile tilapia leathers demonstrated 14.63 N mm^−2^ of traction, 75.11% of elongation, and 50.56 N mm^−1^ of progressive tearing. However, the values obtained by the authors for tensile strength and tearing were higher than those obtained in this experiment. The distribution and organization of the layers of collagen fibers are very important in defining leather strength.

## 5. Conclusion

The flower design of rabbit leather is totally different from that of the fish due to the presence of open hair follicles and pores on the surface of rabbit leather and due to the presence of protective lamellae and insertion of scales on the fish leather. The histological organization of the deep dermis differs between rabbit and fish skin. In rabbits, the deep dermis consists of thick bundles of collagen fibers arranged in various orientations and does not have an organized arrangement of collagen fibers as in fish skin. In fish skin, there are overlapping layers of longitudinal collagen fiber bundles interspersed with a smaller amount of perpendicular fiber bundles, crossing the skin surface (transversely) and binding the fibers together to provide greater strength, as proven by the strength test. Rabbit leather, despite being thicker, has a lower progressive tearing and tensile strength compared to Nile tilapia, but has higher elasticity. Based on the results obtained, it is recommended to use a piece of fabric (lining) together with the fleshy side of the rabbit leather, to increase resistance when used in clothing and footwear, as these products require greater tensile strength. Thus, it minimizes this restriction for the use of rabbit leather, for the purposes mentioned above.

## Figures and Tables

**Table 1:** Average thickness values and determination of the progressive tearing tests of the Nile tilapia and rabbit leather specimens.

Progressive tearing			
Treatment	Thickness (mm)	Tear (N mm^−1^)	Maximum force (N)
Rabbit	1.43 ± 0.22^a^	24.25 ± 4.34^b^	34.08 ± 5.48^b^
Nile tilapia	1.00 ± 0.12^b^	53.85 ± 6.66^a^	54.08 ± 8.92^a^
C.V. (%)^*∗*^	15.35	14.69	17.54
*P* value	<0.0001	<0.0001	<0.0001

Means ± standard deviation; if there are different letters (^a,b^) there is a statistically significant difference through the F test (*P* < 0.05); ^*∗*^C.V. = coefficient of Variation.

**Table 2:** Average tensile and stretching values of the Nile tilapia and rabbit leather specimens.

Traction and stretching				
Treatment	Traction (N mm^−2^)	Stretching (%)	Maximum strength (N)	Deformation (mm)
Rabbit	8.98 ± 2.67^b^	109.97 ± 13.52^a^	120.08 ± 27.33^b^	65.83 ± 8.07^a^
Tilapia	13.52 ± 1.86^a^	78.97 ± 8.40^b^	140.58 ± 24.43^a^	47.83 ± 4.51^b^
C.V. (%)	21.88	13.52	24.92	12.01
*P* value	<0.0001	<0.0001	0.1220	<0.0001

Means ± standard deviation; If there are different letters (^a,b^) there is a statistically significant difference through the F test (*P* < 0.05); ^*∗*^C.V. = coefficient of variation.

## Data Availability

The data used to support the findings of this study are available from the corresponding author upon request.
